# Light correlated color temperature and task switching performance in preschool-age children: Preliminary insights

**DOI:** 10.1371/journal.pone.0202973

**Published:** 2018-08-30

**Authors:** Lauren E. Hartstein, Monique K. LeBourgeois, Neil E. Berthier

**Affiliations:** 1 Department of Psychological and Brain Sciences, University of Massachusetts Amherst, Amherst, MA, United States of America; 2 Lighting Enabled Systems & Applications Engineering Research Center, Rensselaer Polytechnic Institute, Troy, NY, United States of America; 3 Department of Integrative Physiology, University of Colorado Boulder, Boulder, CO, United States of America; University of Minnesota, UNITED STATES

## Abstract

Data from a growing number of experimental studies show that exposure to higher correlated color temperature (CCT) ambient light, containing more blue light, can positively impact alertness and cognitive performance in older children and adults. To date, few if any studies have examined whether light exposure influences cognitive task performance in preschool-age children, who are in the midst of rapid developmental changes in attention and executive function skills. In this study, healthy children aged 4.5–5.5 years (n = 20; 11 females) completed measures of sustained attention and task switching twice while being exposed to LED light set to either 3500K (a lower CCT) or 5000K (a higher CCT). A control group (n = 18; 10 females) completed the tasks twice under only the 3500K lighting condition. Although the lighting condition did not impact performance on the sustained attention task, exposure to the higher CCT light lead to greater improvement in preschool-age children’s task switching performance (F(1,36) = 4.41, p = 0.04). Children in the control group showed a 6.5% increase in task switching accuracy between time points, whereas those in the experimental group improved by 15.2%. Our primary finding–that exposure to light at a higher correlated color temperature leads to greater improvement in task switching performance–indicates that the relationship between the spectral power distribution of light and executive function abilities is present early in cognitive development. These data have implications for designing learning environments and suggest that light may be an important contextual factor in the lives of young children in both the home and the classroom.

## Introduction

A growing body of work demonstrates that the lighting environment can have profound impacts on domains outside of visual perception such as cognition. Multiple studies report positive effects of exposure to short wavelength (blue) light on alertness and cognitive performance [1, 2]. Blue light is thought to impact alertness via stimulation of the intrinsically photosensitive retinal ganglion cells (ipRGCs), a subset of ocular photoreceptors maximally sensitive to light in the blue portion of the visible spectrum [3, 4], with a peak sensitivity at approximately 490nm [5, 6]. When stimulated, the ipRGCs send signals via the retinohypothalamic tract directly into the suprachiasmatic nucleus (SCN), leading to the suppression of melatonin [7, 8]. Over longer periods of light exposure, the signal from the ipRGCs filters out into various regions of the brain, including the cingulate and dorsolateral prefrontal cortices [9], both of which play a major role in attention and cognitive control [10, 11]. Activation of the ipRGCs by blue light has even been seen in blind individuals [12] suggesting the system is largely independent of visual perception.

Previous research findings have demonstrated improved performance on measures of alertness and executive function abilities in adult participants after daytime exposure to higher correlated color temperature (CCT), blue-enriched white light [13–15]. Studies of adolescents offer results that are largely consistent with the adult literature in showing positive effects after daytime exposure to higher CCT light. For example, studies measuring performance on attention tasks found fewer errors committed after exposure to higher CCT light in high school students [16–18]. In young children, however, links between lighting CCT and cognitive performance are limited and less consistent. One study conducted with third graders (average age 8.3 years) found that children exposed to higher CCT light made fewer errors on an attention test compared with children in a control condition [16]. Conversely, another study of third graders using a similar approach found no differences on test performance between children in the different lighting conditions [19]. Rigorous research on light’s effect on cognition in children younger than age eight years is scarce. During the preschool years, children demonstrate pronounced improvements on a variety of cognitive skills that depend on the dorsolateral prefrontal cortex, such as task switching and inhibitory control [20]. Despite these rapid developmental changes in executive function skills, to our knowledge, no study to date has examined how different lighting conditions impact cognition in preschool-age children.

Given the relatively recent discovery of the ipRGCs, a full picture of their development does not exist. Data from animal models, however, suggest that ipRGCs are present and light sensitive from birth, with an active connection to the SCN, suggesting an earlier developmental trajectory than the rods and cones [21,22]. ipRGC’s express the photopigment melanopsin, which has been identified in human eye tissue as early as eight weeks post-conception [23], indicating the foundation for this system is present early in human development. In addition, the pupillary light reflex, initiated by the ipRGCs [24, 25], emerges in preterm infants between 30 and 35 weeks [26]. Taken together, these findings suggest that the blue-light sensitive ipRGCs are well developed by the preschool age.

Surveys of US preschoolers found children’s average time spent outdoors ranges from 63 minutes to 146 minutes a day [27, 28]. Additionally, a recent study of families in a low-income urban community indicates that by age six years, 97% of children had used mobile devices, most starting before age one year, and 75% of children under age four years had their own mobile device [29]. With the prevalence of time spent indoors and children’s increasing access to blue-light emitting mobile devices, young children are being exposed to more artificial light sources than ever before. With these numbers on the rise, it is imperative to understand what role the lighting environment plays in children’s development.

Recent findings indicate that children are highly sensitive to light as measured by melatonin suppression, with children showing significantly greater melatonin suppression in response to light exposure than that measured in adults and adolescents [30–32]. Children’s melatonin response is also influenced by light CCT: Data from recent studies indicate a significant correlation between the CCT of lights in the home and circadian phase [33] as well as greater melatonin suppression in children exposed to light at a higher CCT of 6000K compared with those exposed to a 3000K light condition [34]. In addition, children have significantly larger pupil diameters than adults [32], as well as clearer lenses [35], allowing more blue light to pass into the eye. As such, it is expected that exposure to higher CCT light will have significant impacts on children’s cognitive performance, possibly stronger than that previously seen in adults.

This study will extend foundational knowledge of light’s effect on cognition by examining the effects of daytime exposure to higher CCT light on preschool-age children’s cognitive performance. Participants were tested twice on measures of sustained attention and cognitive flexibility. Sustained attention refers to the ability to devote attention to a single stimulus over an extended period of time [36]. Sustained attention undergoes significant developmental changes between the ages of 4 and 6 years [37] and plays an important role in school achievement, serving as a moderator between general intelligence and GPA, as shown in research findings with high school students [38]. Cognitive flexibility is a key component of executive functions [39], which predicts academic achievement throughout childhood [40–42]. Participants first completed the tasks under light set to a lower CCT at 3500K followed by a higher CCT setting of 5000K. A control group of participants was also tested twice under the 3500K light setting. We hypothesized that preschool-age children exposed to the higher CCT light would show significantly greater improvement on tasks measuring attention and cognitive flexibility than children exposed only to light of a lower CCT. With these data, parents and educators could make empirically-based decisions to create supportive learning environments for young children.

## Methods

### Participants

Forty-five children aged 4.5-to-5.5 years from the greater Amherst, MA area completed a single in-laboratory experimental session lasting approximately one hour. Data were collected across a 12-month period from February 2016 to February 2017. Participants were contacted through e-mail and phone after being identified from state birth records, and parents were asked to confirm their child met the inclusion criteria before the appointment was scheduled. Children were eligible to participate if they were between 4.5–5.5 years old, had no history of psychological or neurological disorders, were not taking any psychotropic medication, and had not traveled across time zones for one month before testing. One participant was excluded for failing to meet the eligibility requirements, two for experimenter error, and four for non-compliance. Thus, the final sample included 38 children (range = 4.5–5.5 years, *M* = 4.91 years, *SD =* 0.26 years; 21 female; 34 Caucasian, 2 Hispanic, 2 mixed-race). Thirty-four children were currently enrolled in preschool, 3 were about to start kindergarten, and 1 was not currently enrolled in school. Children received a small toy as a token of appreciation for their participation. All study procedures were approved by the University of Massachusetts Amherst Institutional Review Board. Written informed consent was obtained from a parent.

### Apparatus and setting

Study settings and procedures were adapted from Hartstein, et al. [14]. Testing took place in a small, windowless laboratory measuring 2.6 m x 2.2 m and 2.3 m tall, with a white ceiling and off-white painted walls. The testing room contained a desk covered with a white sheet. The decision to have the ceiling, walls, and table matte white was to reflect the light from the luminaires without glare. A Dell Inspiron 1501 laptop computer, used to administer the cognitive tasks, was placed on the desk. Study tasks were programmed using E-Prime Version 1.2.

**Fig 1:**
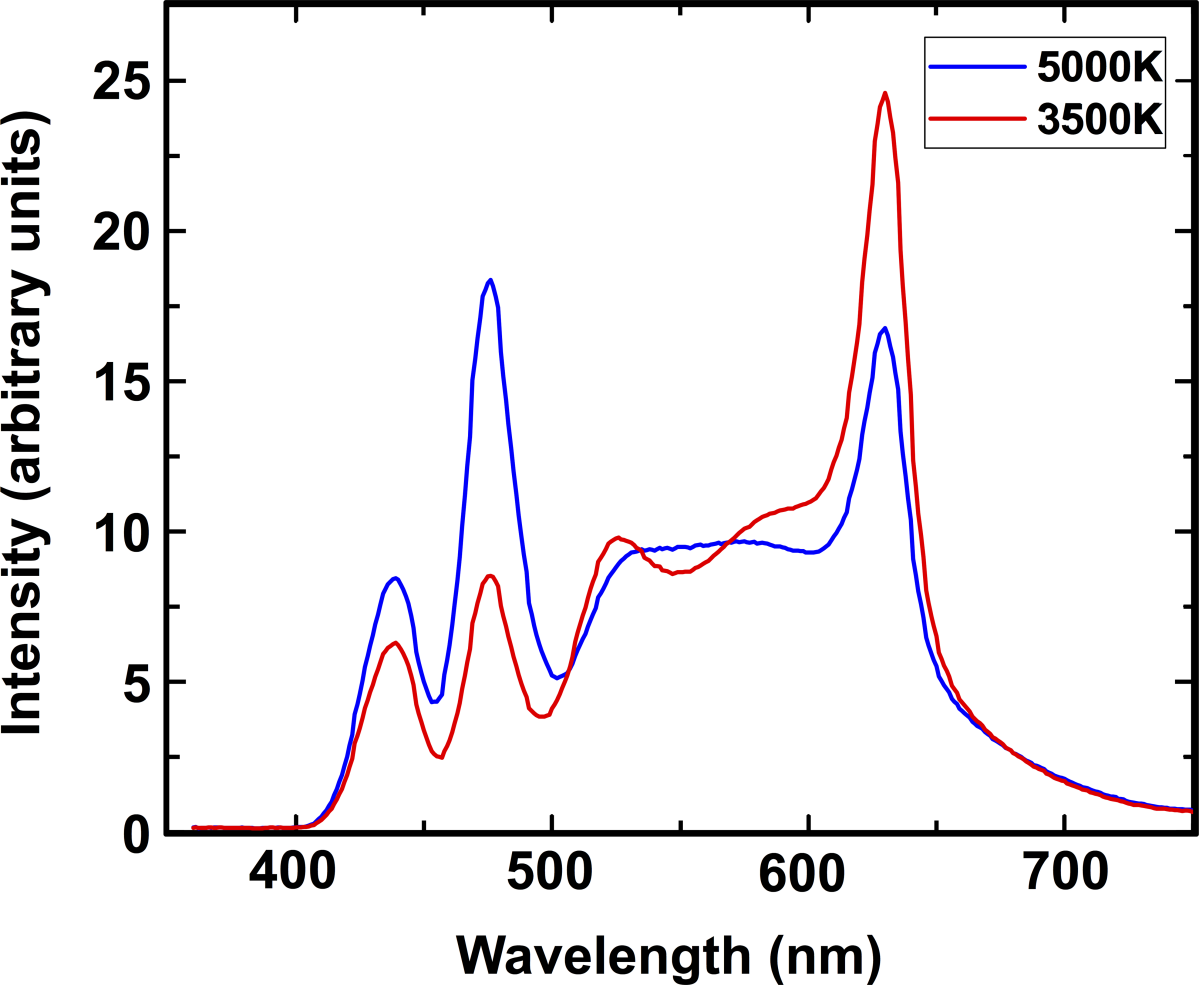
Spectra of the room lights at 3500K and 5000K measured at the source.

Two LED luminaires, centered in the ceiling, were positioned above the desk. The lights were 0.6m x 0.6m (2’ x 2’) CREE LED color tunable fixtures, containing 5 different color channels (red, green, blue, amber, phosphor converted white), mixed together at various levels to create light of different CCTs. For the lower CCT lighting condition, the light was set to 3500K, a common setting chosen for working environments. For the higher CCT lighting condition, the light was set to 5000K. Fig 1 shows the spectra for each of the light settings. The 3500K setting has a peak emission at 630nm. The 5000K setting has a peak emission at 475nm, close to the maximal sensitivity of the ipRGCs.

The illuminance at each participant’s eye level with the lights and laptop both turned on was set to be consistent across lighting conditions and was measured with a LX1330B digital light meter to be approximately 250 lux. Illuminance measurements taken at the workplane facing the light source were 715 lux and 683 lux for the 3500K and 5000K conditions respectively. The color-rendering index, a measure of how well an artificial light source reveals the colors of an object relative to a natural light source [43], was held constant across conditions (CRI = 94 and 93 for the 3500K and 5000K conditions respectively). Although the participants were exposed to light from the laptop as well as the luminaires, the laptop screen constituted only about 10% of the total light reaching the participant’s eye. Furthermore, the laptop screen illuminance was held constant for all participants and across conditions.

Besides looking at the light spectrum as a whole, we can divide the spectrum to calculate effective illuminance, which provides a measures of how much light is impacting each of the five specific types of photoreceptor in the eye (S Cone, M Cone, L Cone, Rod, and ipRGC). Table 1 shows the effective illuminance perceived by each type of photoreceptor when exposed to the two lighting conditions, calculated from the illuminance measurements taken at the workplane [24]. Compared with the baseline setting of 3500K, the 5000K light setting leads to a reduction in stimulation of the L cones, corresponding to the decrease in red light emitted by the light source, and a large increase in stimulation of the S cones and, critically, the ipRGCs, maximally stimulated by light in the blue portion of the visible spectrum. It should be noted, however, that the calculations for effective illuminance were based off of photopigment absorption for an adult observer [24] and values for young children may differ.

**Table 1:** Effective illuminance perceived by photocells for each lighting condition, taken facing the light source.

Photocell	Effective illuminance (lux)	
	3500K	5000K
S Cone (blue)	318.01	519.10
ipRGC	450.59	650.09
Rod	507.32	646.97
M Cone (green)	615.81	665.88
L Cone (red)	713.88	685.97

### Procedure

Participants were randomly assigned to either the control or experimental group before arrival to the testing environment. Informed consent was obtained from the parent while the child participant acclimated to the researchers while playing with some toys. Although the parents were fully informed of the purpose of the study and whether the light was going to be changed, the details of the study were not discussed with the children so that they did not focus on any possible changes in lighting condition. Parents were also asked to answer questions regarding the children’s sleep patterns, including their typical bed time, wake time, and napping habits, as well as how long they slept the previous night. Following informed consent, the participant and his or her parent were brought into the testing room and the participant was seated at the desk in front of the laptop computer. The parent was seated behind the child at the back of the room, out of the child’s line of sight.

During the baseline assessment, the light in the room was set to 3500K for all participants. Participants completed two computer tasks: a Go/No-Go task measuring selective attention [44, 45] and a Hearts and Flowers task, which assesses cognitive flexibility, or the ability to switch between rules.

The order of task presentation was counter-balanced across participants. Following the first completion of the tasks (baseline assessment), participants were briefly taken out of the testing room in order to choose storybooks to read during the subsequent 20-minute adaptation period. While the participants were out of the room, if they were in the experimental condition, the researcher covertly changed the light in the testing room from 3500K to 5000K. For the control condition, the light settings were not changed.

Participants were then brought back into the testing room, where they stayed for the remainder of the study. They then read age-appropriate storybooks with the researcher or their parents for 20 minutes, which served as an adaptation phase during which participants in the experimental condition adjusted to the new lighting environment. Following the adaptation period, participants completed the two computer tasks a second time (test assessment), in the reversed order from which they completed them previously.

#### Go/no-go task

Participants were told that they were going to play a game about going to the zoo [46]. They were shown an image of a cartoon zookeeper and told that “one day, the zookeeper wasn’t paying attention and all the animals escaped from the zoo!” Children were then told that their job was to help the zookeeper catch the escaped animals. In order to do that, they needed to press the right mouse button whenever they saw an animal appear. They were then shown a picture of a monkey and told that the monkey is their friend, who is helping them to catch the animals, so when they see the monkey, they shouldn’t press the button. Children were then asked to repeat the instructions to confirm their comprehension. If the child did not understand, the instructions were repeated until the child demonstrated a sufficient understanding of the instructions. The task was composed of 64 trials, in a semi-random order. The task consisted of 75% “go” trials and 25% “no-go” trials. “Go” trials consisted of images of six different animals: a flamingo, a tiger, a tortoise, a hippopotamus, a zebra, and an antelope.

Each trial image was displayed for 800 ms, separated by a white slide with a fixation cross displayed for 500 ms. Results were scored for accuracy, the percent of correct “no-go” trials out of 16, in which the child successfully inhibited responding to the image of the monkey, as well as reaction time, the latency on correct “go” trials, in which the child correctly “caught” one of the other six animals.

#### Hearts and flowers

**Fig 2:**
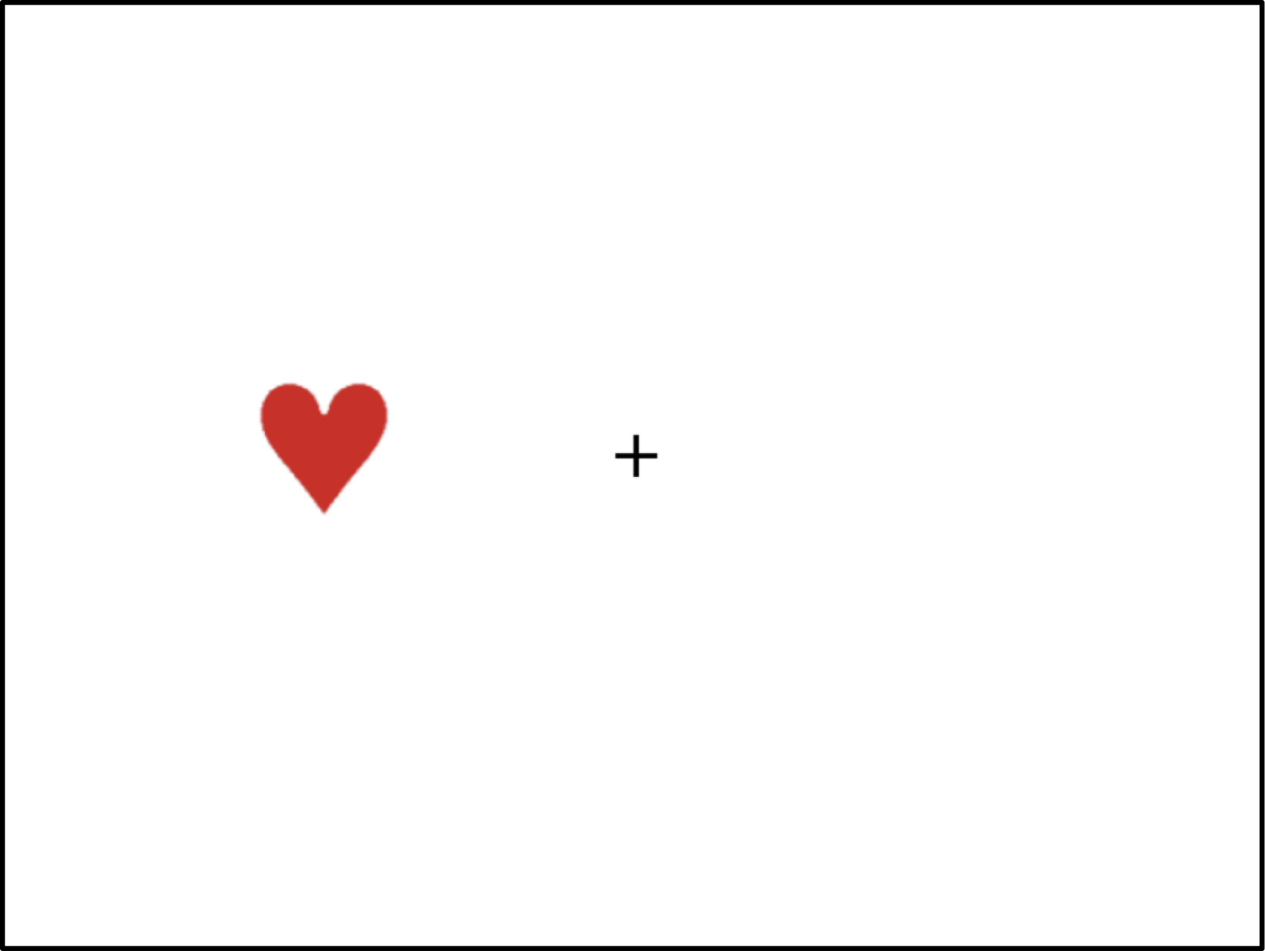
Example of stimulus from Hearts and Flowers task, a measure of task switching ability.

Task components and procedures were adapted from Davidson, et al. [39]. The Hearts and Flowers task consisted of two practice blocks, each with 16 trials, followed by a test block with 40 trials. In each trial, an image of a heart or a flower appeared on either the left or right side of the screen (**Fig 2**). The children were instructed that when they saw a heart, they should press the mouse button that matches the same side where the heart appears on the screen. Alternatively, when they saw a flower, they should press the mouse button that is on the opposite side of where the flower appears on the screen. For example, if the flower appeared on the left, they should press the right mouse button. Children were instructed to press the button corresponding to either the same or opposite side on which the image appears, depending on whether it is a heart or a flower.

The first practice block consisted of only heart trials, the second practice block consisted of only flower trials, and the test block consisted of both types of trials mixed together. The test block consisted of 40 trials, 20 of each type of stimulus, 10 appearing on each side of the screen, presented in a random order. Before each block began, the instructions for that block were reviewed with the child, who was asked to demonstrate which button he or she should press for each possible trial. During each trial, a fixation cross appeared for 500 ms, followed by the trial stimulus. The stimulus appeared onscreen for 2500 ms. Trials were broken down into “no-switch” trials, in which two of the same type of stimulus appeared in a row (i.e. a heart followed by another heart), and “switch” trials, in which the trial followed a trial of the opposite stimulus type (i.e. a heart followed by a flower). Previous findings with adult participants demonstrated significant performance on switch trials following exposure to light set to a higher CCT [14]. Participants’ accuracy (percent correct) and reaction times on correct trials were recorded and analyzed by trial type.

### Analysis

Statistical analyses (S1 Dataset) were performed with SPSS Statistics 21.0 (IBM Corp. Armonk, NY, USA). Independent t-tests were used to examine differences in age, sleep habits, study participation time, and baseline task performance between participants assigned to the control and experimental groups. Data from each task were analyzed with 2-way 2x2 repeated measures ANOVAs to examine differences between the two light conditions (Control, Experimental) and across the two testing time points (Baseline, Test). If the lighting condition impacted participants’ performance over time, we would expect to see significant interactions. Effect sizes for significant results are given as partial-eta squared.

## Results

Table 2 provides a comparison of age, sleep habits, and study time between participants in the control and experimental groups. T-tests revealed that significantly more participants in the control group reported taking daily naps than those in the experimental group, t(30.5) = 2.78, p = 0.01. No significant differences between the two groups were found for parent report of the child’s average bedtime, average wake time, or how long the child slept the night before testing (all p > 0.05), although parents of children in the experimental group reported their children having slept an average of 37 minutes more the previous night than children in the control group. Although participants could not all be tested at the same time of day, the distribution of the study time did not differ significantly between the two groups, t(36) = -1.27, p = 0.21.

**Table 2:** Descriptive statistics, M(SD), for demographics, sleep habits, and study participation time.

	Control (n = 18)	Experimental (n = 20)	Statistics
Sex	10 female	11 female	X^2^ = .11, p = 0.74
Age	4.95yrs (0.28)	4.88yrs (.26)	t(36) = .80, p = 0.43
Bed Time	20:15 (0:44m)	20:11 (0:46m)	t(36) = .25, p = 0.80
Wake Time	06:54 (0:32m)	07:05 (0:47m)	t(36) = -.78, p = 0.44
Hours Slept Previous Night	10.19 (1.29)	10.81 (0.77)	t(27.2) = -1.78, p = 0.09
Daily Naps (%)	55.6	15.0	t(30.5) = 2.78, p = 0.01
Study Time	11:36 (2h:22m)	12:35 (2h:26m)	t(36) = -1.27, p = 0.21

### Go/no-go task

Descriptive statistics for accuracy and reaction time are presented in Table 3. Accuracy was scored as the correct number of “no-go” trials out of 16, in which participants correctly inhibited pressing the mouse button upon seeing the picture of the monkey. Reaction time was calculated in ms for correct “go” trials, in which participants correctly pressed the mouse button in response to a “go” stimulus. Independent t-tests revealed no differences between groups on baseline performance for either accuracy or reaction time (all t < 1.0).

**Table 3:** Descriptive statistics, M(SD), for accuracy (Acc) and reaction time (RT) on the Go/No-Go and Hearts and Flowers tasks.

	Control		Experimental		Interaction Statistics	
	Baseline	Test	Baseline	Test	*F*	*p*
Go/No-Go Acc	78.81(12.13)	80.56(17.00)	78.44(15.13)	80.31(17.69)	0.001	0.98
Go/No-Go RT (ms)	593.93(51.45)	568.38(48.41)	585.46(49.33)	564.18(60.11)	0.06	0.81
Hearts/Flowers Overall Acc	65.69(19.27)	74.03(18.21)	66.50(18.02)	80.13(16.85)	2.65	0.11
Hearts/Flowers Switch Acc	63.44(18.99)	69.89(19.68)	61.67(19.70)	76.90(16.63)	4.41	0.04*
Hearts/Flowers No-Switch Acc	66.59(22.57)	77.08(20.39)	71.19(19.39)	84.06(19.54)	0.43	0.52
Hearts/Flowers Overall RT (ms)	1197.75(357.73)	1118.65(328.18)	1221.47(312.56)	1172.14(238.73)	0.15	0.70
Hearts/Flowers Switch RT (ms)	1323.51(424.29)	1222.85(339.19)	1373.41(360.56)	1284.36(265.26)	0.02	0.90
Hearts/Flowers No-Switch RT (ms)	1092.07(318.92)	1040.04(337.74)	1102.16(282.96)	1066.89(233.99)	0.05	0.82

* *p* < 0.05

Note: Go/No-Go Accuracy was scored as the percent of correct “no-go” trials out of a possible 16. Reaction time was averaged across correct “go” trials. Hearts and Flowers Accuracy was scored as percent of correct trials (calculated separately across all trials and switch trials). Reaction time was averaged across correct trials only. 2x2 ANOVAs were performed comparing Light Condition (Control, Experimental) and Phase (Baseline, Test).

#### Accuracy

Both the control and experimental groups showed only minor improvements between the baseline and test assessments. The analysis indicated no significant main or interaction effects (all F < 1.0).

#### Reaction time

A significant main effect of Phase was found, F(1,36) = 7.14, p = 0.01, η_p_^2^ = 0.17, with participants in both groups showing decreased reaction times from the baseline to test assessments. No significant effects were found for Light Condition, F(1,36) = 0.19, p = 0.67, or Light Condition by Phase interaction, F(1,36) = 0.06, p = 0.81.

### Hearts and flowers task

Only trials in which the participant answered correctly were included in the analyses. Results were analyzed by overall performance across the 40 trials in the mixed block as well as performance on “switch” trials, in which participants had to rapidly switch between the two types of trials. Descriptives for accuracy and reaction time are presented in Table 3, divided into overall performance and performance on “switch” trials. Accuracy was calculated as percent correct. Reaction time is averaged across correct trials. Independent t-tests revealed no differences between groups on the baseline performance across all measures of accuracy and reaction time (all t < 1).

#### Accuracy

Overall accuracy was calculated as the percent of correct trials out of the 40 trials given in the mixed block. A significant main effect of Phase was found, F(1,36) = 45.60, p < 0.001, η_p_^2^ = 0.56, such that participants across both groups showed improvements in accuracy between the baseline and test assessments. Although both groups of participants showed improved accuracy between the baseline and test assessments, the experimental group showed a larger improvement (13.95%) than participants in the control group (8.29%); however, the Light Condition by Phase interaction did not reach significance, F(1,36) = 2.65, p = 0.11. No significant main effect of Light Condition was observed, F(1,36) = 0.37, p = 0.55.

For switch trials, results showed a significant main effect of Phase, F(1, 36) = 26.86, p < 0.001, η_p_^2^ = 0.43, such that participants in both groups showed improvements in accuracy on switch trials between the baseline and test assessments. A significant Light Condition by Phase interaction was found, F(1,36) = 4.41, p = 0.04, η_p_^2^ = 0.11. Similar to the overall trials, although participants in both groups demonstrated improved accuracy on switch trials between time points, participants in the experimental group showed a significantly larger improvement (15.23%) than participants in the control group (6.45%), t(36) = -2.10, p = 0.04, d = 0.68. These results indicate that, following exposure to the higher correlated color temperature light setting, participants in the experimental group showed a significantly larger improvement in their ability to switch between trial types than participants in the control group. No significant main effect of Light Condition was found, F(1,36) = .002, p = 0.97. No significant interaction was found for no-switch trials, F(1,36) = 0.25, p = 0.62.

#### Reaction time

Reaction time was calculated as the average reaction time (in ms) for correct trials across the 40 total trials in the mixed block. Table 3 provides descriptive statistics for each group across the two time points. No significant main effects or interaction effects were observed. For the switch trials, a significant main effect of Phase was found, F(1,36) = 4.44, p = .04, with participants in both groups showing faster reaction times in the test assessment compared with the baseline. Data indicated no significant effects for Light Condition, F(1,36) = 0.29, p = 0.60, or Light Condition by Phase interaction, F(1,36) = 0.02, p = 0.90. No significant main effects or interactions were found for performance on no-switch trials.

## Discussion

To our knowledge, this is the first experimental study to examine whether exposure to higher CCT light (5000K) improves cognitive performance in healthy preschool-age children compared with exposure to light set to a lower CCT (3500K), utilizing a tightly-controlled mixed model design. As hypothesized, participants demonstrated significantly greater improvement in their performance on a task measuring their ability to switch between tasks following exposure to the experimental lighting condition; however, we found no effect of the lighting condition on performance on the sustained attention task. Children in the experimental group demonstrated a 15.2% increase in switch accuracy between time points, as measured by performance on the Hearts and Flowers task, compared with children in the control group whose accuracy increased an average of 6.5%. In addition, the main effect of Phase for reaction time on switch trials suggests that children in the experimental group gained significantly greater accuracy on switch trials than participants in the control group without sacrificing speed, consistent with results from previous work measuring the relationship between young children’s speed and accuracy on measures of executive function [47]. These results extend previous findings with adult participants that exposure to higher CCT light improves performance on a task switching task [13, 14]. Results are discussed in the context of the relationship between the built environment and development, as well as emerging trends in technology.

Our results provide important evidence demonstrating that the relationship between light spectral power distribution and cognitive task performance is observable in children as young as 4.5 years of age. The preschool-age period is one of rapid developmental change, as children begin to fine-tune multiple dimensions of executive function skills, including working memory, inhibitory control, and cognitive flexibility. Cognitive flexibility, however, develops more slowly across early childhood than other dimensions of executive functions. One study testing children aged 4 to 13 years on a task switching task found that 13-year-olds did not perform at adult levels [39]; however, the current results suggest that exposure to a bluer light source can help children effectively switch between rules and exercise greater cognitive flexibility. Subsequent work should explore how exposure to higher CCT light at this sensitive developmental period impacts not only short-term changes but also long-term development of these skills.

Although our results provide preliminary evidence of the relationship between light CCT and cognitive task performance in preschool-age children, the mechanisms underlying these effects are not clear. It is known that stimulation of the ipRGC leads to activation of the SCN, regulating the circadian system [7], before filtering out to regions associated with cognitive control. Higher CCT light, containing more blue light and more closely resembling the natural daylight environment, stimulates these cells and tells the body’s internal clock that it is time to be awake and alert. Adults exposed to higher CCT light in the daytime frequently report higher subjective alertness and decreased feelings of sleepiness [15]. In addition, previous findings have demonstrated that insufficient sleep negatively impacts children’s cognitive task performance [48]. Increased alertness and decreased sleepiness in children exposed to the higher CCT lighting condition would support the observed improvements in cognitive task performance. Although accurate self-report of alertness and sleepiness are difficult to obtain in young children, future studies should use subjective as well as physiological measures of alertness to better understand the specific processes underlying these effects. Additionally, a previous study found that higher CCT light leads to better near acuity in children aged 10–11 years [49]. It is therefore possible that improved near acuity in preschool-age children in the experimental group contributed to their performance on the hearts and flowers task.

Environmental contexts can play an important role in children’s learning [50, 51]; however, current research on light CCT and its impact on cognition in early childhood is limited. Several color-tunable fixtures are already being marketed to schools as a means of influencing student behavior in the classroom. These fixtures allow teachers to select the lighting environment from pre-programmed options to suit the demands of the classroom activity and promote learning. With the implementation of such devices in classrooms and the drastic rise of children’s exposure to blue-light emitting electronic devices [29, 52], it is crucial to understand and quantify the impacts of daytime artificial light exposure on multiple childhood developmental domains. The present results have the capacity to aid in informing the construction of optimal learning environments for young children in homes and schools, including utilization of light at a higher CCT to enhance children’s executive function abilities from a young age. Previous research findings have identified children’s performance on executive function tasks as a significant predictor of mathematical ability [40], as well as associated with scholastic achievements in math, English, and science [41, 42]. Future work, however, is needed to clarify whether improvements on measures of executive function as a result of the lighting environment translate to improvements in scholastic performance.

Contrary to our hypothesis, we found no differences between participants in each lighting condition on sustained attention. This contrasts previous findings with adults demonstrating positive impacts of higher CCT light on go/no-go task performance [14, 44]. Accuracy on the task was high, with participants averaging approximately 80% correct on no-go trials, and both the control and experimental groups showed only minor improvement in accuracy from the baseline to test assessments, suggesting participants were performing near ceiling level. This finding suggests that the task parameters may not have been challenging enough to elicit any differences as a function of the lighting condition, which is consistent with previous findings from adult participants showing that the impact of the lighting condition is influenced by the task difficulty [53]. Furthermore, the task used in this study consisted of only 16 “no-go” trials, creating the possibility that the task was not sensitive enough to detect small group differences. Thus, an important next step will be to explore whether light CCT impacts children’s performance on more complex attention tasks or tasks comprised of a greater number of trials.

Although this study presents important findings on the impact of light on preschool-age children’s cognitive abilities using a tightly-controlled experimental design, the limitations of this work are important to note. Only one study was conducted to test our hypotheses and our sample was relatively small. As such, the findings of the present study should be considered preliminary. The alerting effects of light in adults are impacted by an individual’s prior light history [54]. Because no data on children’s light history were collected, the influence of previous exposure cannot be discounted. Children’s sleep patterns and sleep duration were obtained through parental report, which may be subject to reporter bias and should be interpreted with caution [55]. Furthermore, although our results demonstrated improvement in task switching ability under the higher CCT lighting condition, the study did not include a condition in which the light was changed to a lower CCT, leaving open the possibility that the present results would be obtained by any change in lighting; however, the repeated findings of the positive effects of higher CCT on cognitive performance in adults suggest this is unlikely to be the case [13–15, 44]. Lastly, the generalizability of the results is limited, as they were obtained in a controlled laboratory environment with healthy, neurotypical children. Future, well-controlled fieldwork is needed to determine if the same effects of the lighting condition would be observed in everyday settings, such as the classroom or home using real world scholastic activities.

In summary, these findings add to a growing body of work demonstrating the impacts of light outside of visual perception. Our primary finding–that exposure to light at a higher CCT leads to greater improvements in a measure of preschool-age children’s cognitive flexibility provides preliminary evidence suggesting the early emergence of the relationship between the lighting environment and cognitive task performance. These results highlight the importance of further research about these effects in the context of learning. Such data may inform parents and educators on the impacts of the environment on cognitive development in the early years of life.

## Supporting information

S1 Dataset Subject demographic information and task performance. This Excel spreadsheet contains the data used for analyses in the present study. The columns in the dataset are ID Number, Condition (Control or Experimental), Age (in months), Gender (M or F), Study Time, Average Bed Time, Average Wake Time, Hours Slept Previous Night, Naps (daily naps, Y or N), Go/No-Go Accuracy Baseline (out of 16), Go/No-Go Accuracy Test (out of 16), Go/No-Go Reaction Time Baseline (ms), Go/No-Go Reaction Time Test (ms), Hearts and Flowers Overall Accuracy Baseline (% correct), Hearts and Flowers Overall Accuracy Test (% correct), Hearts and Flowers Switch Accuracy Baseline (% correct), Hearts and Flowers Switch Accuracy Test (% correct), Hearts and Flowers No-Switch Accuracy Baseline (% correct), Hearts and Flowers No-Switch Accuracy Test (% correct), Hearts and Flowers Overall Reaction Time Baseline (ms), Hearts and Flowers Overall Reaction Time Test (ms), Hearts and Flowers Switch Reaction Time Baseline (ms), Hearts and Flowers Switch Reaction Time Test (ms), Hearts and Flowers No-Switch Reaction Time Baseline (ms), Hearts and Flowers No-Switch Reaction Time Test (ms).
(XLSX)
