# Correction: Light correlated color temperature and task switching performance in preschool-age children: Preliminary insights

**DOI:** 10.1371/journal.pone.0205542

**Published:** 2018-10-04

**Authors:** The PLOS ONE Staff

The following information is missing from the Funding section: Publication of this article was funded by the University of Colorado Boulder Libraries Open Access Fund.

The publisher apologizes for the error.
